# Enzyme Inhibitory Properties, Antioxidant Activities, and Phytochemical Profile of Three Medicinal Plants from Turkey

**DOI:** 10.1155/2015/410675

**Published:** 2015-12-20

**Authors:** Gokhan Zengin, Gokalp Ozmen Guler, Abdurrahman Aktumsek, Ramazan Ceylan, Carene Marie Nancy Picot, M. Fawzi Mahomoodally

**Affiliations:** ^1^Department of Biology, Science Faculty, Selçuk University Campus, 42250 Konya, Turkey; ^2^Department of Biological Education, Ahmet Keleşoğlu Education Faculty, Necmettin Erbakan University, 42090 Konya, Turkey; ^3^Department of Health Sciences, Faculty of Science, University of Mauritius, 230 Réduit, Mauritius

## Abstract

We aimed to investigate the inhibitory potential of three medicinal plants (*Hedysarum varium*,* Onobrychis hypargyrea*, and* Vicia truncatula*) from Turkey against key enzymes involved in human pathologies, namely, diabetes (*α*-amylase and *α*-glucosidase), neurodegenerative disorders (tyrosinase, acetylcholinesterase, and butyrylcholinesterase), and hyperpigmentation (tyrosinase). The antioxidant potential, phenolic and flavonoid content of ethyl acetate, and methanolic and aqueous extracts were investigated using* in vitro* assays. The total antioxidant capacity (TAC), *β*-carotene/linoleic acid bleaching activity, 1,1-diphenyl-2-picrylhydrazyl free radical (DPPH^•^), 2,2-azino-bis(3-ethylbenzothiazoline-6-sulfonic acid) (ABTS^•+^), cupric ion reducing antioxidant capacity (CUPRAC), ferric reducing antioxidant power (FRAP), and metal chelating activity on ferrous ions were used to evaluate the antioxidant capabilities of the extracts. The half-maximal inhibitory concentrations (IC_50_) of the extracts on cholinesterase, tyrosinase, and *α*-amylase were significantly higher than the references, galantamine, kojic acid, and acarbose, respectively. The half-maximal effective concentrations (EC_50_) of the extracts on TAC, CUPRAC, and FRAP were significantly higher than trolox. The phenol and flavonoid contents of the plant extracts were in the range 20.90 ± 0.190–83.25 ± 0.914 mg gallic acid equivalent/g extract and 1.45 ± 0.200–39.71 ± 0.092 mg rutin equivalent/g extract, respectively. The plants were found to possess moderate antioxidant capacities and interesting inhibitory action against key enzymes.

## 1. Introduction

Turkey has been described as one of the countries which has the richest floral biodiversity worldwide [1]. Indeed, this is due to its unique geographical location, climatic conditions, and geomorphological characteristics [2, 3]. Approximately, 10,500 species have been identified in Turkey and 30% were found to be endemic [2]. The relatively high rate of endemism in Turkey provides an indication of the richest biodiversity in this area [1].

Herbal medicinal systems, knowledge, and practices have been transmitted through the ages. For centuries, medicinal plants were the only resources available for the treatment of several diseases which plagued humanity. In fact, many of today's drugs have been derived from medicinal plants [1]. Additionally, the World Health Organisation has reported that 80% of the world's population relies on herbal medicine for primary health care [2]. Recently, several ethnobotanical studies have reported the widespread usage of plants for curative purposes among the local Turkish people [2, 4, 5]. However, to the best of our knowledge, several medicinal plants used as folk Turkish medicine have not received scientific attention yet.

In Turkey, the Fabaceae family is the second largest family after Asteraceae and the most economically important family after Poaceae [6, 7]. Ethnobotanical studies have reported that important taxa from Fabaceae family have been used in folk medicine. For instance,* Onobrychis gracilis* is commonly used for cold and flu [8];* Vicia faba* is used to treat gastrovascular disorders [9];* Vicia cracca* subsp.* stenophylla* is used as an anticough agent [10];* Vicia ervilia* is used to treat diabetes [11]. In the present investigation, 3 Fabaceae species, namely,* Hedysarum varium, Onobrychis hypargyrea*, and* Vicia truncatula*, were evaluated for their possible antioxidant activities and inhibitory action on cholinesterase, tyrosinase, *α*-amylase, and *α*-glucosidase.

## 2. Materials and Methods

### 2.1. Plant Material and Extraction

*Hedysarum varium* (Hv) (38°1′49.25′′N, 32°30′10.91′′S) was collected from Konya, and* Onobrychis hypargyrea* (Oh) (40°18′30.00′′N, 32°58′39.00′′S) and* Vicia truncatula* (Vt) (40°27′17.00′′N, 32°37′27.00′′S) were collected from Ankara. The plants were identified by Dr. Murad Aydin Sanda, the senior taxonomist of the Department of Biology, Selçuk University, Konya, Turkey, and voucher specimens were deposited at the herbarium of the laboratory. The aerial parts of the plants were dried at room temperature. Air-dried samples (10 g) were macerated in 200 mL solvent (ethyl acetate (EA), methanol (MeOH), or water (Aq)) at room temperature for 24 h. The extracts were concentrated under reduced pressure and organic extracts were dissolved in methanol while the aqueous extract was dissolved in water.

### 2.2. Quantification of Phenolic Compounds

#### 2.2.1. Determination of Total Phenol Content

The total phenol content was determined as described by Slinkard and Singleton [12] with slight modifications. Briefly, 0.25 mL plant extract was mixed with a tenfold diluted Folin-Ciocalteu reagent solution and the mixture was shaken vigorously. After 3 min, 0.75 mL sodium carbonate solution (1%) was added to the mixture and was allowed to react for 2 h at room temperature. The absorbance was then read at 760 nm. The total phenol content was expressed as mg gallic acid equivalents (GAE) per g crude extract using a gallic acid standard curve.

#### 2.2.2. Determination of Total Flavonoid Content

The total flavonoid content was determined following the method described by Berk et al. [13]. Briefly, 1 mL aluminium trichloride (2%) solution in methanol was added to 1 mL plant extract. The absorbance of the mixture was read at 415 nm after 10 min incubation at room temperature. The total flavonoid content was expressed as mg rutin equivalents (RE) per g crude extract using a rutin standard curve.

### 2.3. Determination of Antioxidant Activities

#### 2.3.1. Total Antioxidant Capacity (TAC)

The reduction of molybdenum(VI) to molybdenum(V) by the plant extracts was used to assess the total antioxidant capacity following the method described by Berk et al. [13] with slight modifications. An aliquot of plant extract (0.3 mL) was combined with 3 mL of reagent solution (0.6 M sulfuric acid, 28 mM sodium phosphate, and 4 mM ammonium molybdate). The absorbance was read at 695 nm after 90 min incubation at 95°C. EC_50_, that is, the effective concentration at which the absorbance was 0.5, was calculated for the plant extracts and trolox.

#### 2.3.2. *β*-Carotene/Linoleic Acid Bleaching Activity

The antilipid peroxidation capacities of the plant extracts were measured by *β*-carotene/linoleic acid bleaching [14]. A stock solution of *β*-carotene and linoleic acid was prepared from 0.5 mg *β*-carotene dissolved in 1 mL chloroform, 25 *μ*L linoleic acid, and 200 mg Tween 40. The chloroform was completely evaporated using a vacuum evaporator and 100 mL of oxygenated distilled water was added to the residual mixture. The mixture was shaken vigorously and 1.5 mL of this mixture was added to 0.5 mL plant extract and the absorbance at time 0 was measured at 490 nm. The absorbance was monitored at regular intervals, that is, at 30, 60, 90, and 120 min. The bleaching rate (*R*) of  *β*-carotene was calculated according to

(1) $$\begin{array}{c} R=\left[\;\frac{\ln\left(a/b\right)}{t}\;\right], \end{array}$$

where ln represents natural log, *a* is the absorbance at time 0, and *b* is the absorbance at time *t* (30, 60, 90, and 120 min). The antioxidant activity (AA) was calculated in terms of percentage inhibition relative to the control from

(2) $$\begin{array}{c} \text{AA}=\left[\;\frac{\left(R_{Control}-R_{Sample}\right)}{R_{Control}}\;\right]\times 100. \end{array}$$

IC_50_, that is, the concentration of plant extract/trolox required to scavenge 50% of linoleate, was then determined.

#### 2.3.3. 1,1-Diphenyl-2-picrylhydrazyl (DPPH) Free Radical Scavenging Assay

The effect of the plant extracts on DPPH radical was assessed according to the method described by Sarikurkcu [15]. Briefly, 1 mL of plant extract was added to 4 mL DPPH solution (0.004%) in methanol. The absorbance was measured at 517 nm after 30 min incubation at room temperature in the dark. The radical scavenging activity was calculated as follows: % inhibition = [(Abs_blank_ − Abs_sample_)/Abs_blank_] × 100, where Abs_blank_ is absorbance of the blank and Abs_sample_ is absorbance of the sample. Trolox is used as a positive control and IC_50_ was then determined.

#### 2.3.4. 2,2-Azino-bis(3-ethylbenzothiazoline-6-sulfonic acid) (ABTS) Radical Cation Scavenging Activity

The scavenging activity of the plant extracts on ABTS^∙+^ radical was measured according to the method of Re et al. [16] with slight modifications. ABTS^∙+^ radical was produced by reacting 7 mM ABTS solution with 2.45 mM potassium persulfate. The mixture was allowed to stand for 12 to 16 h in the dark at room temperature. The resulting ABTS solution was diluted with methanol and adjusted to absorbance of 0.700 ± 0.02 at 734 nm. Plant extract (1 mL) was added to ABTS solution (2 mL) and after 30 min incubation at room temperature the absorbance was measured at 734 nm. The radical scavenging activity was calculated as follows: % inhibition = [(Abs_blank_ − Abs_sample_)/Abs_blank_] × 100, where Abs_blank_ is absorbance of the blank and Abs_sample_ is absorbance of the sample. Trolox is used as a positive control and IC_50_ was then calculated.

#### 2.3.5. Cupric Ion Reducing Antioxidant Capacity (CUPRAC) Assay

The cupric ion reducing antioxidant capacity (CUPRAC) was determined according to the method described by Apak et al. [17]. Plant extract (0.5 mL) was added to the reaction mixture containing 10 mM copper chloride (1 mL), 7.5 mM neocuproine (1 mL), and 1 M ammonium acetate buffer at pH 7 (1 mL). The absorbance was read at 450 nm after 30 min incubation at room temperature. EC_50_ was determined for each plant extract.

#### 2.3.6. Ferric Reducing Antioxidant Power (FRAP) Assay

The FRAP assay was carried out as described by Aktumsek et al. [18] with slight modifications. Plant extract (0.1 mL) was added to FRAP reagent solution (2 mL) containing 0.3 M acetate buffer, pH 3.6, 10 mM 2,4,6-tris(2-pyridyl)-s-triazine (TPTZ) in 40 mM HCl, and 20 mM ferric chloride in a ratio of 10 : 1 : 1 (v/v/v). The absorbance was then measured at 593 nm after 30 min incubation at room temperature. EC_50_ of the plant extract was then determined.

#### 2.3.7. Metal Chelating Activity on Ferrous Ions

The metal chelating activities of the plant extracts on ferrous ions were determined by the method described by Aktumsek et al. [18]. Briefly, 2 mL of plant extract was added to 0.05 mL 2 mM iron chloride. The reaction was initiated by the addition of 0.2 mL 5 mM ferrozine. The absorbance was read at 562 nm after 10 min incubation at room temperature. The chelating activity was calculated as follows: % inhibition = [(Abs_blank_ − Abs_sample_)/Abs_blank_] × 100, where Abs_blank_ is absorbance of the blank and Abs_sample_ is absorbance of the sample. EDTA is used as a positive control and IC_50_ was calculated.

### 2.4. Determination of Cholinesterase, Tyrosinase, *α*-Amylase, and *α*-Glucosidase Activity

#### 2.4.1. Cholinesterase Inhibition Assay

Cholinesterase inhibitory activity was measured using Ellman's method as previously reported by Aktumsek et al. [18] with slight modifications. The plant extract (50 *μ*L) was mixed with dithiobisnitro-benzoate (DTNB) (125 *μ*L) and cholinesterase solution (25 *μ*L) in Tris-HCl buffer (pH 8.0) in a 96-well microplate. The reaction was initiated by the addition of 25 *μ*L of acetylthiocholine iodide or butyrylthiocholine chloride. The absorbance was read at 405 nm after 10 min incubation at room temperature. The anticholinesterase activity was calculated as follows: % inhibition = [(Abs_blank_ − Abs_sample_)/Abs_blank_] × 100, where Abs_blank_ is absorbance of the blank and Abs_sample_ is absorbance of the sample. Galantamine is used as a positive control and IC_50_ value was determined.

#### 2.4.2. Tyrosinase Inhibition Assay

Tyrosinase inhibitory activity was measured using the modified dopachrome method previously described by Orhan et al. [19] with slight modifications. Plant extract (25 *μ*L) was mixed with tyrosinase solution (40 *μ*L) and phosphate buffer (pH 6.8) (100 *μ*L) in a 96-well microplate and incubated for 15 min at 37°C. L-DOPA (40 *μ*L) was then added to the mixture to initiate the reaction. The absorbance was read at 492 nm after 10 min incubation at 37°C. The percentage inhibition of tyrosinase was calculated as follows: % inhibition = [(Abs_blank_ − Abs_sample_)/Abs_blank_] × 100, where Abs_blank_ is absorbance of the blank and Abs_sample_ is absorbance of the sample. Kojic acid is used as a positive control and IC_50_ was calculated.

#### 2.4.3. *α*-Amylase Inhibition Assay

*α*-Amylase inhibitory activity was performed using the Caraway-Somogyi iodine-potassium iodide method [20] with some modifications. Briefly, plant extract (25 *μ*L) was mixed with *α*-amylase solution (50 *μ*L) in phosphate buffer (pH 6.9) with 6 mM sodium chloride in a 96-well microplate and incubated for 10 min at 37°C. The reaction was initiated by the addition of 0.05% starch solution (50 *μ*L). The reaction mixture was incubated for 10 min at 37°C. The reaction was then stopped by the addition of 1 M HCl (25 *μ*L), followed by addition of the iodine-potassium iodide solution (100 *μ*L). The absorbance was measured at 630 nm. The percentage inhibition of *α*-amylase was calculated as follows: % inhibition = [(Abs_blank_ − Abs_sample_)/Abs_blank_] × 100, where Abs_blank_ is absorbance of the blank and Abs_sample_ is absorbance of the sample. Acarbose is used as a positive control and IC_50_ was determined.

#### 2.4.4. *α*-Glucosidase Inhibition Assay

*α*-Glucosidase inhibitory activity was performed following the previous method described by Palanisamy et al. [21] with some modifications. Plant extract (50 *μ*L) was mixed with glutathione (50 *μ*L), *α*-glucosidase solution (50 *μ*L) in phosphate buffer (pH 6.8), and PNPG (50 *μ*L) in a 96-well microplate and incubated for 15 min at 37°C. The reaction was stopped by the addition of 0.2 M sodium carbonate (50 *μ*L) and the absorbance was read at 400 nm. The percentage inhibition of *α*-glucosidase was calculated as follows: % inhibition = [(Abs_blank_ − Abs_sample_)/Abs_blank_] × 100, where Abs_blank_ is absorbance of the blank and Abs_sample_ is absorbance of the sample. Acarbose is used as a positive control and IC_50_ was determined.

### 2.5. Statistical Analysis

The experiments were carried out in triplicate. The results are expressed as mean ± standard deviation (SD). The differences between the different extracts were analyzed using one-way analysis of variance (ANOVA) followed by Tukey's honestly significant difference post hoc test with *α* = 0.05 using SPSS v. 14.0.

## 3. Results

### 3.1. Quantification of Phenolic Compounds

The total phenol and flavonoid content of the plant extracts are summarised in Table 1. Oh extracts yielded higher phenol content in the following order: OhEA > OhMeOH > OhAq. On the other hand, it was observed that the flavonoid content of the plant extracts varied in the following order: MeOH > Aq > EA. The methanolic and aqueous extracts of Hv showed the highest flavonoid content.

### 3.2. Determination of Antioxidant Activities

Table 2 summarises the reducing power and radical scavenging and metal chelating capacities of Hv, Oh, and Vt extracts. It was found that the plant extracts showed variable radical scavenging capabilities on DPPH^∙^ and ABTS^∙+^. Methanolic extracts of Hv and Oh and aqueous extract of Oh (IC_50_: 0.30 ± 0.005, 0.29 ± 0.002, and 0.27 ± 0.001 mg/mL, resp.) significantly (*P* < 0.05) scavenged DPPH^∙^ as compared to the positive control trolox (IC_50_: 0.31 ± 0.003 mg/mL). On the other hand, it was observed that the plant extracts scavenged ABTS^∙+^ but were significantly (*P* < 0.05) less active than trolox (IC_50_: 0.18 ± 0.004 mg/mL). Likewise, the plant extracts showed low chelating activity on ferrous ions. Ethyl acetate extracts of Hv and Vt (IC_50_: 1.07 ± 0.006 and 1.05 ± 0.001 mg/mL, resp.) showed potent *β*-carotene bleaching capacities as compared to trolox (IC_50_: 1.10 ± 0.004 mg/mL). Additionally, it was noted that the plant extracts exhibited variable reducing potential. However, as shown in Table 2, none of the plant extracts exhibited reducing activity which was significantly (*P* < 0.05) lower than trolox.

It was observed that the plant extracts exhibited variable inhibitory effects on cholinesterases (acetyl cholinesterase and butyryl cholinesterase), tyrosinase, *α*-amylase, and *α*-glucosidase (Table 3). The plant extracts were significantly (*P* < 0.05) less active than the positive controls galantamine, kojic acid, and acarbose against cholinesterases, tyrosinase, and *α*-amylase, respectively. However, Hv extracts (IC_50_: 3.77 ± 0.016, 2.88 ± 0.051, and 5.18 ± 0.078 mg/mL for ethyl acetate, methanolic, and aqueous extract, resp.), methanolic and aqueous extracts of Oh (IC_50_: 3.89 ± 0.097 and 5.86 ± 0.050 mg/mL, resp.), and ethyl acetate extract of Vt (IC_50_: 2.74 ± 0.044 mg/mL) significantly (*P* < 0.05) inhibited *α*-glucosidase as compared to acarbose (IC_50_: 6.67 ± 0.200 mg/mL).

## 4. Discussion

The use of plant-based products for the management and treatment of diseases is gaining much momentum from both scientific and consumer perspectives. Indeed, herbal therapies have been used for curative purposes since the dawn of civilisation. The relentless efforts for wellbeing and to combat diseases have guided scientists as well as health care providers towards safer and natural alternatives such as medicinal plants. Currently, there is a renewed interest in natural inhibitors from plant-based medicines to modulate physiological effects of enzymes linked to several pathologies such as diabetes, obesity, neurodegenerative diseases, and inflammation, amongst others. The present study has endeavoured to investigate the possible inhibitory effects of three medicinal plants to modulate key enzymes involved in diabetes (*α*-amylase and *α*-glucosidase), neurodegenerative disorders (tyrosinase, acetylcholinesterase, and butyrylcholinesterase), and melanogenesis (tyrosinase).

Diabetes is a chronic disease characterised by elevated blood sugar level which leads to the onset of serious health complications such as cardiovascular problems, nephropathy, and neuropathy [22]. The inhibition of *α*-amylase and *α*-glucosidase which are involved in the hydrolysis of sugars* in vivo* has been an important strategy for the management of diabetes thereby lowering postprandial glucose level. Inhibitors of *α*-glucosidase delay the breaking down of carbohydrate in the gut and decrease postprandial blood glucose peak in diabetic patients. Synthetic oral hypoglycaemic agents such as acarbose, miglitol, and voglibose are currently used for the treatment of diabetes [23]. However, their side effects, such as abdominal discomforts and flatulence, have guided research towards safer and more effective alternatives notably from natural sources [24]. In the present study, the plant extracts showed inhibition against both *α*-amylase and *α*-glucosidase. Additionally, it was noted that the plant extracts were potent inhibitors of *α*-glucosidase and the methanolic and aqueous extracts of Hv and Oh showed significantly lower IC_50_ values than acarbose and therefore can be potentially useful as an effective therapy for postprandial hyperglycemia with minimal side effects. This is in line with report of Picot et al. [25] who reported natural *α*-glucosidase inhibitors from plants to have strong inhibition towards the activity of the enzyme compared to acarbose.

Plant extracts from the present study were found to inhibit acetylcholinesterase although their inhibitory action was less potent than the known inhibitor galantamine. It was also found that some of the plant extracts showed inhibition against another cholinesterase enzyme, butyrylcholinesterase. Inhibition of cholinesterases, the key enzymes in the breakdown of acetylcholine, is considered one of the treatment strategies against several neurological disorders. The inhibition of cholinesterases leads to an increase in the concentration of acetylcholine in the brain which subsequently results in an increase in communication between the brain nerve cells [22, 26]. Indeed, both acetylcholinesterase and butyrylcholinesterase inhibitors have been key targets for the treatment of neurodegenerative disorders such as Alzheimer's disease [27, 28]. Cholinesterase inhibitors constitute, to date, the most effective approach to treat the cognitive symptoms of neurological disorders. Hence, plants studied in the present study can be of therapeutic utility both on cognitive performances and on the quality of life in these patients.

Tyrosinase is a key enzyme responsible for the hydroxylation of tyrosine to L-DOPA and its subsequent oxidation to dopaquinone [29]. Dopaquinone and its derivatives produced via the biosynthesis of melanin by tyrosinase are thought to play a pivotal role in the degeneration of nigrostriatal dopaminergic neurons in Parkinson's disease [30]. Tyrosinase inhibitors have attracted much interest due to the key role of tyrosinase in mammalian melanogenesis [31]. Inhibitors of the tyrosinase enzyme, such as kojic acid (by-product in the fermentation of malting rice, produced naturally by several species of fungi such as* Aspergillus oryzae*), azelaic acid (isolated from wheat, rye, and barley and produced naturally by* Malassezia furfur*), and arbutin (extracted from bearberry plants), have been utilised in the pharmaceutical and cosmetic industry for their potential of thwarting the excessive production of melanin. However, controversies still persist in the literature concerning their safety and efficacy [31, 32]. Interestingly, plant extracts evaluated in the present study were found to inhibit tyrosinase activity but at higher concentration than the known inhibitor kojic acid.

The variation in activity of the plant extracts against these enzymes might be explained based on the complex composition and potential synergistic effect(s) of individual phytochemicals present in each sample. Interestingly, we found varying concentration of phenolics and flavonoids in extracts of these plants. Previously, it has been reported that inhibitory activity of plant extracts might be due to the presence of several phytochemicals such as flavonoids, saponins, and tannins. Additionally, studies on *α*-amylase and *α*-glucosidase inhibitors isolated from medicinal plants suggest that several potential inhibitors belong to flavonoid class which has features of inhibiting metabolic enzymes. Recently, it has been shown that phenolics play a role in mediating amylase inhibition and therefore have potential to contribute to the management of type 2 diabetes [33].

Free radicals are known to play a pivotal role in the onset and exacerbation of several pathologies [34]. By counteracting these free radicals, antioxidants help in preserving good health. Indeed, phytochemicals have received much interest owing to their molecular structure which consists of hydroxyl groups on aromatic rings and this has been associated with their functionality as oxidant scavengers [35]. Phytochemicals act by inhibiting oxidative chain reactions at cellular level thereby increasing their therapeutic efficacy [36]. In the present study, the phenolic content of the plant extracts was estimated using the Folin-Ciocalteau method. This method is rapid and simple but also measures various interfering nonphenolic compounds such as ascorbic acid, thiol, and nitrogen containing compounds [37]. Flavonoids are the major class of phenolic compounds and are known to exhibit strong antioxidant activities [38, 39]. Interestingly, in the present study, it was observed that Oh extract showed high phenolic and flavonoid content.

Various assays were employed to study the antioxidant potential of the extracts of Hv, Oh, and Vt. The gross antioxidant capacities of the plant extracts were determined using two methods, namely, the TAC and *β*-carotene bleaching assays. The TAC assay is based on the reduction of Mo(IV) to Mo(V) by antioxidants and the formation of green phosphate/Mo(V) compound [36]. On the other hand, the ability of the plant extracts to scavenge linoleate-derived free radicals and thus prevent *β*-carotene bleaching was also investigated [40]. It was observed that Oh extracts actively reduced Mo(IV) to Mo(V) but their EC_50_ values were significantly higher than the standard trolox. It was also noted that ethyl acetate extracts showed IC_50_ significantly lower than trolox for the *β*-carotene bleaching assay. This was associated with the “polar paradox theory” which suggests that nonpolar antioxidants are more effective in relatively nonpolar systems [24]. The high phenolic and flavonoid content of Oh were linked to the observed gross antioxidant capacity.

The reducing power of plant extracts is regarded as an indication of their antioxidant capacities [41]. FRAP and CUPRAC were used to assess the reductive potentials of the plant extracts. The plant extracts exerted variable reducing potentials thereby suggesting that phenolic compounds acted as reductones. Reductones are thought to exert antioxidant action by donating a hydrogen atom thus breaking the chain reaction [42]. Additionally, it was reported that reductones react with peroxide precursors thereby preventing peroxide formation [43].

The free radical quenching potential of the plant extracts was determined using two nitrogen-centered radicals, namely, DPPH^∙^ and ABTS^∙+^. DPPH^∙^ is a stable dark purple free radical which turns into a yellow stable diamagnetic molecule upon reaction with antioxidants [44]. On the other hand, ABTS^∙+^ is a blue radical cation which is converted into a colourless form in the presence of a hydrogen donor [42]. Results from the present study have demonstrated that the plant extracts showed good abilities to quench both DPPH^∙^ and ABTS^∙+^. The ability of the plant extracts to quench DPPH^∙^ and ABTS^∙+^ was related to the observed high phenol content.

One of the most important mechanisms of action of antioxidants involves the chelation of prooxidant metals such as iron. Iron promotes oxidation by acting as catalyst of free radical chain reactions [45]. The chelation of iron by phytochemicals decreases its prooxidant effect through the stabilisation of its oxidised form [40]. Indeed, the plant extracts were found to chelate iron but were less potent than EDTA.

## 5. Conclusion

Data gathered from the present investigation demonstrated that Hv, Oh, and Vt possessed antioxidant capabilities and also exhibited inhibitory potential against cholinesterase, tyrosinase, *α*-amylase, and *α*-glucosidase* in vitro*. Furthermore, to date, no such scientific information on these plants has been gathered. However, it was observed that the antioxidant capacities and *α*-amylase, cholinesterase, and tyrosinase inhibitory activities of the plant extracts were less potent than the controls. Thus, it might be argued that the plants possessed moderate antioxidant and enzyme inhibitory properties. Further studies are needed for the identification of bioactive constituents for the determination of molecular mechanisms involved in antioxidant and enzymatic activities of these plant extracts.

## Figures and Tables

**Table 1:** Total phenol and flavonoid content of the plant extracts.

Plant extracts	Total phenol content (mg GAE/g extract)	Total flavonoid content (mg RE/g extract)
HvEA	20.96 ± 1.291	2.41 ± 0.205
HvMeOH	45.11 ± 1.399	39.71 ± 0.092
HvAq	37.97 ± 1.033	34.53 ± 2.001

OhEA	83.25 ± 0.914	9.92 ± 0.030
OhMeOH	73.20 ± 0.756	27.00 ± 0.544
OhAq	69.38 ± 0.992	25.20 ± 0.088

VtEA	27.19 ± 1.283	1.45 ± 0.200
VtMeOH	20.90 ± 0.190	22.45 ± 0.325
VtAq	25.86 ± 0.085	8.67 ± 0.109

Hv: *Hedysarum varium*; Oh: *Onobrychis hypargyrea*; Vt: *Vicia truncatula*; EA: ethyl acetate extract; MeOH: methanolic extract; Aq: aqueous extract.

**Table 2:** Reducing power, metal chelation activity, and radical scavenging potential of the plant extracts.

Plant extracts/positive controls	IC_50_ (mg/mL)				EC_50_ (mg/mL)		
	DPPH^•^	ABTS^•+^	Metal chelating	*β*-Carotene/linoleic acid bleaching activity	TAC	CUPRAC	FRAP
HvEA	6.27 ± 1.523^*∗∗*^	11.81 ± 0.682^*∗∗*^	NA	1.07 ± 0.006^*∗*^	1.18 ± 0.013^*∗∗*^	3.01 ± 0.279^*∗∗*^	1.98 ± 0.107^*∗∗*^
HvMeOH	0.30 ± 0.005^*∗*^	2.75 ± 0.004^*∗∗*^	51.54 ± 4.795^*∗∗*^	1.10 ± 0.009^*∗∗*^	1.54 ± 0.045^*∗∗*^	0.69 ± 0.031^*∗∗*^	0.44 ± 0.007^*∗∗*^
HvAq	0.35 ± 0.014^*∗∗*^	1.50 ± 0.039^*∗∗*^	1.40 ± 0.010^*∗∗*^	1.10 ± 0.006^*∗∗*^	2.85 ± 0.001^*∗∗*^	0.80 ± 0.008^*∗∗*^	0.43 ± 0.007^*∗∗*^

OhEA	0.31 ± 0.003^*∗∗*^	1.20 ± 0.007^*∗∗*^	NA	1.14 ± 0.008^*∗∗*^	0.90 ± 0.014^*∗∗*^	0.81 ± 0.021^*∗∗*^	0.34 ± 0.005^*∗∗*^
OhMeOH	0.29 ± 0.002^*∗*^	1.27 ± 0.007^*∗∗*^	47.27 ± 1.255^*∗∗*^	1.12 ± 0.070^*∗∗*^	1.09 ± 0.021^*∗∗*^	0.89 ± 0.056^*∗∗*^	0.39 ± 0.003^*∗∗*^
OhAq	0.27 ± 0.001^*∗*^	1.22 ± 0.014^*∗∗*^	16.79 ± 0.456^*∗∗*^	1.06 ± 0.007^*∗∗*^	1.44 ± 0.101^*∗∗*^	0.80 ± 0.011^*∗∗*^	0.37 ± 0.004^*∗∗*^

VtEA	1.15 ± 0.002^*∗∗*^	5.91 ± 0.083^*∗∗*^	10.80 ± 1.453^*∗∗*^	1.05 ± 0.001^*∗*^	1.13 ± 0.004^*∗∗*^	1.58 ± 0.276^*∗∗*^	1.33 ± 0.061^*∗∗*^
VtMeOH	0.84 ± 0.060^*∗∗*^	6.23 ± 0.035^*∗∗*^	32.35 ± 2.493^*∗∗*^	1.12 ± 0.008^*∗∗*^	2.48 ± 0.035^*∗∗*^	1.44 ± 0.098^*∗∗*^	1.16 ± 0.026^*∗∗*^
VtAq	0.47 ± 0.004^*∗∗*^	5.50 ± 0.252^*∗∗*^	20.62 ± 0.060^*∗∗*^	1.12 ± 0.003^*∗∗*^	3.35 ± 0.008^*∗∗*^	1.13 ± 0.015^*∗∗*^	0.65 ± 0.009^*∗∗*^

Trolox	0.31 ± 0.003	0.18 ± 0.004	ND	1.10 ± 0.004	0.59 ± 0.006	0.11 ± 0.008	0.05 ± 0.003
EDTA	ND	ND	0.04 ± 0.001	ND	ND	ND	ND

Hv: *Hedysarum varium*; Oh: *Onobrychis hypargyrea*; Vt: *Vicia truncatula*; EA: ethyl acetate extract; MeOH: methanolic extract; Aq: aqueous extract. ^*∗*^Values significantly (*P* < 0.05) lower than the positive control. ^*∗∗*^Values significantly (*P* < 0.05) higher than the positive control. NA: not active; ND: not determined.

**Table 3:** Inhibition concentration of the plant extracts on cholinesterases, tyrosinase, *α*-amylase, and *α*-glucosidase.

Plant extracts/positive controls	IC_50_ (mg/mL)				
	Cholinesterases		Tyrosinase	*α*-Amylase	*α*-Glucosidase
	Acetylcholinesterase	Butyrylcholinesterase			
HvEA	1.44 ± 0.001^*∗∗*^	3.29 ± 0.018^*∗∗*^	2.63 ± 0.035^*∗∗*^	3.65 ± 0.188^*∗∗*^	3.77 ± 0.016^*∗*^
HvMeOH	1.50 ± 0.044^*∗∗*^	NA	2.50 ± 0.014^*∗∗*^	5.59 ± 0.191^*∗∗*^	2.88 ± 0.051^*∗*^
HvAq	9.22 ± 0.527^*∗∗*^	NA	3.46 ± 0.012^*∗∗*^	13.39 ± 0.219^*∗∗*^	5.18 ± 0.078^*∗*^

OhEA	1.46 ± 0.016^*∗∗*^	3.81 ± 0.252^*∗∗*^	4.30 ± 0.057^*∗∗*^	4.92 ± 0.335^*∗∗*^	20.95 ± 0.581^*∗∗*^
OhMeOH	1.63 ± 0.018^*∗∗*^	NA	3.50 ± 0.069^*∗∗*^	5.51 ± 0.141^*∗∗*^	3.89 ± 0.097^*∗*^
OhAq	4.46 ± 0.024^*∗∗*^	NA	21.76 ± 1.357^*∗∗*^	11.84 ± 0.465^*∗∗*^	5.86 ± 0.050^*∗*^

VtEA	1.57 ± 0.003^*∗∗*^	3.15 ± 0.052^*∗∗*^	3.21 ± 0.012^*∗∗*^	2.07 ± 0.095^*∗∗*^	2.74 ± 0.044^*∗*^
VtMEOH	1.60 ± 0.008^*∗∗*^	NA	2.26 ± 0.010^*∗∗*^	4.21 ± 0.180^*∗∗*^	8.68 ± 0.214^*∗∗*^
VtAq	5.55 ± 0.080^*∗∗*^	NA	4.41 ± 0.145^*∗∗*^	13.74 ± 0.514^*∗∗*^	8.31 ± 0.355^*∗∗*^

Galantamine	0.002 ± 0.000	0.002 ± 0.001	ND	ND	ND
Kojic acid	ND	ND	0.14 ± 0.001	ND	ND
Acarbose	ND	ND	ND	1.00 ± 0.023	6.67 ± 0.200

Hv: *Hedysarum varium*; Oh: *Onobrychis hypargyrea*; Vt: *Vicia truncatula*; EA: ethyl acetate extract; MeOH: methanolic extract; Aq: aqueous extract. ^*∗*^Values significantly (*P* < 0.05) lower than the positive control. ^*∗∗*^Values significantly (*P* < 0.05) higher than the positive control.
